# Measuring disease activity in adults with systemic lupus erythematosus: the challenges of administrative burden and responsiveness to patient concerns in clinical research

**DOI:** 10.1186/s13075-015-0702-6

**Published:** 2015-07-20

**Authors:** Jamal Mikdashi, Ola Nived

**Affiliations:** Division of Rheumatology and Clinical Immunology, University of Maryland School of Medicine, 10 South Pine Street, Suite 834, Baltimore, MD 21201 USA; Department of Rheumatology, Institution of Clinical Sciences, Lund University Hosptial, SE-221 85 Lund, Sweden

## Abstract

Measuring lupus disease activity accurately remains a challenging and demanding task given the complex multi-system nature of lupus, an illness known for its variability between patients and within the same patient over time. Many have attempted to define what disease activity means and how it should be measured, and several instruments were devised for a standardized assessment of disease activity and outcome domains in clinical research. Several of these measuring tools have been able to detect clinical improvement and have demonstrated adequate reliability, validity, and sensitivity to change in observational studies, and some were found to be useful in randomized controlled trials. However, several failed clinical trials have confronted these metrics, as they were not intended for clinical trials. The Outcome Measures Rheumatology group and the US Food and Drug Administration have recommended using measures of disease activity, cumulative organ damage, health-related quality of life, and adverse events as outcomes of interest. Composite responder indices that determine disease global improvement, ensure no significant worsening in unaffected organ systems, and include a physician’s global assessment have been used in randomized clinical trials. Yet unmet therapeutic needs were further challenged by the complex content and psychometric information of the updated instruments, including increased administrative burden associated with demanding training and cost of instruments, and small effect size associated with responsiveness to patient concerns. Nevertheless, with the progress of novel targeted therapy, refining the disease activity metrics is essential. Selection of the disease activity endpoints which is a defining aspect of clinical trial design must be tailored to the outcome of interest and measured by a reliably rated scale characterized by minimal administrative burden. An optimal scale should be simple and practical and incorporate elements of patient concerns.

Measurement of disease activity in systemic lupus erythematosus (SLE) is central to clinical research when evaluating clinical outcomes, comparing meaningful differences among SLE patient groups, and assessing disease activity longitudinally for observational and clinical trials. Several reliable and validated instruments have been available since the early 1980s, and some updated measures are now being used in clinical trials for classifying and monitoring groups of patients and gauging responses to a new drug [1–8]. The administrative burden of the current versions of these tools, their psychometric properties, and how much they are responsive to patient concerns still have not been well addressed.

The complex nature of SLE with fluctuating levels of disease activity involving one or multiple organs, which may vary between patients and within the same patient over time, continues to challenge SLE investigators. The absence of a ‘gold standard’ for defining disease activity, and the diverse psychometric properties of each proposed scale contributes to the difficulty when refining these tools. The inter-rater variability in the assessment of disease activity confronts even an experienced evaluator or a well-trained investigator. The predictability of detecting a substantial meaningful change is far more challenging than the complexity encountered when using the instrument itself [9, 10]. The administrative burden of the disease activity measure with its intricate psychometric properties needs to be taken into consideration when choosing an instrument applicable in a particular research or clinical setting. The administrative burden expands beyond the knowledge about the instrument itself to include the preparedness and skillfulness of the assessor, the mode of administration, the time required to complete the instrument, and the complexity of scoring. Furthermore, the varied length of the scales (number of items and scoring scale), number of patients included, or disease severity of patients under study influence the performance across proposed instruments and weigh into the administrative burden through required advanced training and familiarity of the instrument.

The Outcome Measures Rheumatology group and the US Food and Drug Administration (FDA) had recommended using measures of disease activity, cumulative organ damage, health-related quality of life (HRQOL), and adverse events as outcomes of interest [11]. Patient-reported outcome measures broadly classified as descriptive, discriminative, evaluative, or predictive or a combination of these are being incorporated in clinical trials yet still await further adaptation and validation to reflect an accurate measure of any intervention. Responsiveness remains a key element of the psychometric properties of any instrument. It is pivotal to identify and validate appropriate global, disease-specific, and perhaps organ-specific health-related outcomes for clinical research.

This article reviews the commonly used disease activity tools and discusses: (a) strengths and weaknesses of each of the disease activity measures and responder indices, with an emphasis on the psychometric properties; (b) the administrative burden and cost of training; and (c) how much each tool is capable of capturing responsiveness to patient concerns. A summary of several disease activity indices is depicted in Table 1. The article concludes with recommendations on the optimal disease activity measures and responder index research tool along with a set of practical suggestions for developing a research agenda for detecting meaningful outcome in lupus research.

**Table 1:** Systemic lupus erythematosus disease activity measurements and their psychometric properties, administrative burden, and responsiveness to patient concerns

SLE disease activity measure	Psychometric properties	Administrative burden	Responsiveness to patient care
BILAG and BILAG-2004	Reliable, valid, and sensitive to small change over time	A computer is needed.	Responsive
		Laboratory studies are required.	Fibromyalgia syndrome may complicate the assessment of lupus disease activity.
	Strength: Disease activity and severity are incorporated.	Time: 50 minutes	
		Training is required.	
		Cost: $$$	
	Weakness: Formal training is essential for optimal performance.	commercial/academic	
SLAM and SLAM-R	Reliable, valid, sensitive, and responsive to change over time	A physician is to complete history and physical.	Highly responsive
	Strength: includes both disease activity and severity	Laboratory studies are needed.	
		Time: 15 minutes	
	Weakness: subjective scoring by patients	Training is required.	
		Cost: $	
		commercial/academic	
SLEDAI, SELENA-SLEDAI	Reliable, valid, sensitive, and responsive to change over time	A physician is to complete history and physical.	Responsive
SLEDAI-2 K			
	Strength: practical, most commonly used for clinical and research purposes	Laboratory studies are needed. (There are no immunologic tests for the Mexican version.)	
	Weakness: The SLEDAI versions do not capture improving or worsening and do not include severity within an organ system.	Time: 10 minutes	
		Training is required.	
		Cost: $	
		commercial/academic	
SLE Responder Index (SRI)	Reliable and valid	A physician is to complete history and physical.	Modest responsiveness
	Strength: SRI-50 is superior to the SLEDAI-2 K in identifying patients with 50 % or greater improvement.		
SRI-50		Laboratory studies are needed.	
		Time: 15 minutes	
		Training is required.	
	Weakness: The SRI may miss the signal toward improvement.	Cost: $$	
		commercial/academic	
Organ-specific outcome measure	Reliable and valid	A physician is to complete history and physical.	Responsive
	Strength: focused response criteria		
Lupus nephritis outcome measure	Weakness: Defining the optimal endpoint of organ-specific response criteria is needed.	Laboratory studies are needed.	
		Time: 30 minutes	
CLASI	Long-term outcome studies are essential.	Training is required.	
		Cost: $$	
		commercial/academic	
The BILAG-Based Composite Lupus Assessment (BICLA)	Reliable and valid	A physician is to complete history and physical.	Responsive
	Strength: BICLA criteria require a stringent response in all body systems that are involved at baseline and require that there be no new flares in the remaining body system.		
		Laboratory studies are needed.	
		Time: 50 minutes	
		Training is required.	
		Cost: $$$	
		commercial/academic	
	Weakness: Formal training is essential for optimal performance.		
SELENA-SLEDAI Flare Index (SFI)	Reliable and valid	A physician is to complete history and physical.	Responsive
	Strength: The revised SFI suggests specific clinical manifestations for each organ system and categorizes flares into mild, moderate, and severe on the basis of the treatment decision.		
		Laboratory studies are needed.	
		Time: 20 minutes	
		Training is required.	
		Cost: $$	
		commercial/academic	
	Weakness: Training is essential for optimal performance.		
BILAG-2004 flare index	Reliable and valid	A physician is to complete history and physical.	Moderate responsiveness
	Strength: A strict criterion is needed to define flare.		
		Laboratory studies are needed.	
	Weakness: Scoring of worsening in the renal and hematologic domains is not yet available.	Time: 50 minutes	
		Training is required.	
		Cost: $$$	
		commercial/academic	
Lupus Foundation flare	Strength: The flare must be considered clinically significant by the assessor.	A physician is to complete history and physical.	Responsive
	Weakness: The emphasis is on clinical judgment in determining flare significance.	Laboratory studies are needed.	
		Time: 15 minutes	
		Training is required.	
		Cost: $	
	A strict intention to treat is required.	commercial/academic	

BILAG, British Isles Lupus Assessment Group; CLASI, Cutaneous Lupus Erythematosus Disease Area and Severity Index; SELENA-SLEDAI, Safety of Estrogens in Lupus National Assessment-Systemic Lupus Erythematosus Disease Activity Index; SLAM, Systemic Lupus Activity Measure; SLAM-R, Systemic Lupus Activity Measure-Revised; SLE, systemic lupus erythematosus; SLEDAI, Systemic Lupus Erythematosus Disease Activity Index; SLEDAI-2 K, Systemic Lupus Erythematosus Disease Activity Index 2000; SRI-50, SLEDAI-2000 Responder Index 50.

## Major disease activity measures

### British Isles Lupus Assessment Group (BILAG) index and BILAG-2004

The BILAG index, an organ-based transitional activity instrument, provides disease activity scorings across eight organ systems on an ordinal scale (A to E) based on the physician’s intention-to-treat premise [3]. The original version was published in 1988, and the updated version (BILAG-2004) was published in 2005. In the revised index, the original section of vasculitis was removed and two systems were added: ophthalmic and abdominal.

The BILAG-2004 index categorizes disease activity into five different levels from A to E. Grade A represents very active disease likely necessitating immunosuppressive drugs and/or a prednisolone (or equivalent) dose of more than 20 mg daily or high-dose anticoagulation. Grade B represents moderate disease activity requiring a lower dose of corticosteroids, topical steroids, topical immunosuppressive drugs, anti-malarials, or non-steroidal anti-inflammatory drugs. Grade C indicates mild stable disease, and grade D implies no disease activity but suggests the system had previously been affected. Grade E indicates no current or previous disease activity.

BILAG records disease activity occurring over the past 4 weeks. The BILAG-2004 index covers 97 items, and the classic BILAG index contains 86 items. Each question is answered as 0 = not present, 1 = improving, 2 = same, 3 = worse, or 4 = new. The BILAG-2004 system tally provides a disease activity measure that scores longitudinally and is clinically meaningful and easier to analyze in comparison with multiple categorical variables. It has three components (systems with active/worsening disease, systems with improving disease and systems with persistent minimal or no activity). This system has expected associations with change in therapy.

This allows not only detection of changes across different organs but also differentiation of major from minor improvement or (where relevant) deterioration, combining the simplicity of numerical scoring with the clinical intuitiveness of the BILAG-2004 categorical scoring [12]. The British Lupus Integrated Prospective System (BLIPS) is a computerized program that calculates the BILAG scores [3].

#### Psychometric information

The classic BILAG and BILAG-2004 have been found to be reliable, valid, and sensitive to change over time and have correlated with other disease activity measures (in particular, the Systemic Lupus Erythematosus Disease Activity Index, or SLEDAI) [13, 14]. Good reliability (intraclass correlation coefficient (ICC) of more than 0.60), high levels of physician agreement (σ_physician_/σ_patient_ of less than 0.40), and inter-rater reliability of the index with overall ICCs of 0.45 (95 % confidence interval (CI) 0.31 to 0.58) and 0.67 (95 % CI 0.54 to 0.76) have been demonstrated [15]. The overall sensitivity of the index has been determined at 81 %, specificity at 81.9 %, positive predictive value at 56.8 %, and negative predictive value at 93.6 %. Construct and criterion validity have been verified. The BILAG-2004 systems tally requires further validation.

##### Strength

The BILAG-2004 index score incorporates the important element of change in disease state with time. It is sensitive to small changes and distinguishes between disease activity and disease severity. It shows disease activity in individual systems ‘at a glance’ rather than combining them into a global score. Despite the complex calculations, the score is quick to conduct, especially when calculated by a computer, and is only minimally dependent on the particular clinician carrying out the procedure. The numerical scoring system facilitates comparisons with global indices by converting the assessments so that ‘A’ = 12 points, ‘B’ = 8 points, ‘C’ = 1 point, and ‘D/E’ = 0 points [16].

##### Weakness

Formal training of raters and a well-defined glossary are essential to ensure the optimal performance and achieve a valid registration of the index. Despite the high physician agreement in almost all systems, inter-rater validity continues to be superior among the BILAG group compared with other trained researchers.

##### Administrative burden

BILAG is completed by a physician. A computer program is needed to calculate categorical or numerical scoring. In addition to the time spent on complete history and physical examination, BILAG requires up to 50 minutes to administer, and the instrument cannot be scored until laboratory results are available, and this may take a few days. There is no cost to use the BILAG instrument unless the computerized version is needed; the cost then depends upon type of usage (commercial/academic).

##### Responsiveness to patient concerns

Major clinical responses by the BILAG index are a BILAG C score or better at 6 months with no new BILAG A or B scores and the maintenance of response with no new BILAG A or B scores between 6 and 12 months. It should be noted that sleep disorders, depression and fibromyalgia may confound the assessment of lupus disease activity when using the BILAG. With the exception of the mucocutaneous, hematologic, and renal domains, a significant relationship of the individual BILAG component scoring with Medical Outcome Study Short Form SF20+ measured global assessment of patient well-being and health status has been demonstrated.

### Systemic lupus activity measure

The Systemic Lupus Activity Measure (SLAM) index, published in 1988 and revised in 1991, measures global disease activity within the previous month. It was developed on the basis of domain sampling theory. Items chosen for the scale represent those manifestations that occur more frequently, can be graded, and can be operationally defined and reliably rated [17].

Systemic Lupus Activity Measure-Revised (SLAM-R) includes 23 clinical manifestations in nine organs/systems and seven laboratory features and has a possible range of 0 to 81; a score of at least 7 is considered clinically important because it is associated with a probability of initiating therapy in more than 50 % of cases. Each organ item may score 0 to 3 points if any of the said organ’s

- > clinical manifestations were present within the previous month
- > (severity incorporated into higher score per item). Most items can score a maximum of 3 points. Few items can score a maximum of 1 point. The laboratory category can score a maximum of 21 points.

#### Psychometric information

The SLAM-R index has been found to be reliable and valid and to have an excellent sensitivity and responsiveness to change over time [18]. The SLAM index correlates with other disease activity measures, including the BILAG and SLEDAI. The correlation between the SLAM-R scores, the physician’s global assessment, anti-double-stranded DNA, and C3 and C4 were statistically significant, ranging from −0.29 to 0.87.

The reliability of SLAM was demonstrated with an inter-rater reliability and an inter-visit reliability of 0.86 and 0.73, respectively, and findings for the SLAM-R were similar (0.78 and 0.85, respectively). The validity of the index was shown with a significant correlation between the SLAM and the other scales with an average range of 0.9 to 1.0. Convergent validity was demonstrated with an average range of 0.5 to 0.8 across instruments.

##### Strength

The SLAM index includes both dimensions: disease activity and disease severity. It gives equal weighting to mild and serious organ disease activity without considering the significance of the organ involved.

##### Weakness

One of the disadvantages of the SLAM index is that many of its items are subjective, and much of the scoring relies on the reporting of symptoms by the patients. SLAM may also have some difficulty in distinguishing a change, in particular when scoring minimally active disease items versus damage.

##### Administrative burden

A physician is to complete the questionnaire, which is available in paper format or as part of the BLIPS software program. The scoring is simple additive. The maximum score is 81 points. Judgment as to whether manifestations (laboratory or otherwise) are due to lupus is needed.

A complete history and physical examination are also needed. It can take up to 15 minutes to complete the form. Training is needed to develop consensus on subjective components of the index, especially in multi-center studies. There is no cost to use unless the computerized version is needed; the cost then depends upon type of usage (commercial/academic). There is a modest cost to complete laboratory tests.

##### Responsiveness to patient concerns

This index has a high sensitivity to change and responsiveness when the patient’s global assessment is considered to be the standard. The SLAM correlates with several aspects of the patient’s perception of health, as evaluated with the 36-Item Short Form Health Survey (SF-36).

### Systemic lupus erythematosus disease activity index and its versions

The SLEDAI is a global index that was developed and introduced in 1985 as a clinical index for the assessment of lupus disease activity in the preceding 10 days. It consists of 24 weighted clinical and laboratory variables of nine organ systems. This instrument was derived by consensus among experts in rheumatology followed by application of regression models to assign relative weights to each parameter. SLEDAI was modeled on the basis of clinician global judgment. The scores of the descriptors range from 1 to 8, and the total possible score for all 24 descriptors is 105.

### Safety of Estrogens in Lupus National Assessment study-SLEDAI

A modified version of the SLEDAI (SELENA-SLEDAI) was devised for use in the Safety of Estrogens in Lupus National Assessment (SELENA) study. A glossary was added, and the scoring was modified to account for persistent active disease in some descriptors (rash, mucosal ulcers, and alopecia), which were previously not scored unless they were new or recurrent.

In the SELENA-SLEDAI, researchers accepted the presence of either the objective or subjective findings to score the descriptor as present [19]. The SELENA-SLEDAI version awaits rigorous validation with other measures related to disease activity in SLE.

### SLEDAI-2000

SLEDAI-2000 (SLEDAI-2 K) was introduced in 2002 as a measure of global disease activity. SLEDAI-2 K is a modification of the original SLEDAI to allow the documentation of persistent disease activity in the descriptors: rash, alopecia, mucosal ulcers, and proteinuria. SLEDAI-2 K has been validated against the classic SLEDAI, and proven to be sensitive to change over time. SLEDAI is a strong predictor of mortality in SLE [20].

### SLEDAI-2 K (30-day)

A 30-day extension of the SLEDAI-2 K was then tested and demonstrated to be equivalent to the original 10-day version [21, 22]. Descriptors of SLEDAI-2 K are documented as present or absent. Each of the descriptors has a weighted score, and the total score of SLEDAI-2 K is the sum of all 24 descriptor scores. The total SLEDAI-2 K score falls between 0 and 105. A score of 6 is considered clinically important and affects the decision to treat with a probability of initiating therapy in more than 50 % of cases. Meaningful improvement is best defined as a reduction in SLEDAI-2 K of 4.

#### Psychometric information

The SLEDAI has demonstrated validity, reliability, and sensitivity to change in several observational studies. Lupus disease activity measured by SLEDAI has been a major determinant of damage accrual and is highly predictive of mortality within a 6-month period. The reliability of the original SLEDAI was verified with an inter-rater correlation ranging from 0.61 to 0.80 [2]. The reliability of the SLEDAI-2 K was demonstrated with an agreement for each of the items between 81.7 % and 100 % [3]. The SLEDAI validity was verified with an ICC of 0.79 [2]. The SLEDAI-2 K was validated against the SLEDAI with a high correlation between both indices (r = 0.97, *P* = 0.0001) [20].

The SLEDAI sensitivity and responsiveness to change have been shown in comparative studies with the SLAM, BILAG, and European Consensus Lupus Activity Measurements. The sensitivity to change was estimated to be the smallest for the SLEDAI; the standardized response means were 0.48 when the physician global assessment was used as the standard and −0.01 when the patient global assessment was used [10].

##### Strength

All versions are validated and used by lupus researchers for clinical and research purposes. The practical applicability of SLEDAI in clinical settings, its ease of administration, and its simplicity in scoring are fundamental properties. SLEDAI-2 K is one of the most commonly used global disease activity measures in longitudinal observational studies and clinical trials.

##### Weakness

The SLEDAI versions do not capture improving or worsening, do not include severity within an organ system, and are less sensitive to change when compared with other instruments. SLEDAI-2 K is a global index that generates a total score reflecting an overall disease activity but is capable of measuring disease activity in each of the nine organ systems if required. The use of SLEDAI as a single determinant of flare or worsening remains limited since worsening of pre-existing symptoms and less-than-complete remission of such symptoms (even with significant improvement) do not change the SLEDAI score. The SELENA-SLEDAI did provide a separate flare index.

##### Administrative burden

SLEDAI must be completed by a physician. It has a simple additive scoring system and may take up to 10 minutes to complete. A complete history and physical examination are needed. The instrument cannot be scored until laboratory results, including immunological parameters, are available, and this may take a few days. The Mexican modification of the SLEDAI, a simplified version without the immunologic test, makes the index cheaper to administer. There is no cost to use unless the computerized version is needed; the cost then depends upon type of usage (commercial/academic) [23].

##### Responsiveness to patient concerns

Disease activity as measured by SLEDAI does not significantly predict self-reported levels of fatigue [24]. However, across five randomized controlled trials in SLE, lower HRQOL scores at baseline were highly correlated with higher disease activity scores by SLEDAI or SELENA-SLEDAI or both [25].

## Composite indices

### The SLE responder index

The SLE Responder Index (SRI) is a composite outcome that incorporates a modification of SELENA-SLEDAI, BILAG, and a 3-cm visual analog scale of physician-rated disease activity (PGA) to determine patient improvement [26]. The SRI was derived following *post hoc* analysis of data from a phase II belimumab study in SLE to identify subjects with a meaningful clinical improvement in disease activity in response to treatment. The SRI defines a responder as a patient whose disease course fulfils all of the following: (1) at least a 4-point reduction in SELENA-SLEDAI score; (2) no new BILAG A (severe disease activity) or not more than one new BILAG B (moderate disease activity) organ domain score; and (3) no deterioration from baseline in the PGA by at least 0.3 points (or 10 % of 3-point visual analog scale) [27].

### SLEDAI-2000 responder index 50

The SLEDAI-2000 Responder Index 50 (SRI-50) comprises the 24 SLEDAI-2 K descriptors, covering nine organ systems, and generates a total score that reflects disease activity over the previous 30 days as does SLEDAI-2 K. Each of the SRI-50 descriptors identifies at least 50 % improvement which generates a score for the corresponding descriptor [28].

#### Psychometric information

The SRI-50 has been shown to be reliable, valid, and superior to SLEDAI-2 K in detecting partial clinical improvement (at least 50 %) between visits. The average intra-rater reliability values of SLEDAI-2 K, SRI-50, and PGA were 0.99, 0.98, and 0.90, respectively [29]. The SRI-50 has been validated and has shown sensitivity response prospectively at 6 and 12 months and retrospectively at 10 years [30, 31].

##### Strength

SRI-50 has been proven to be superior to the SLEDAI-2 K in identifying patients with 50 % or greater improvement. SRI-50 might improve and facilitate the identification of responders in longitudinal research studies.

##### Weakness

The SRI uses SLEDAI to determine global improvement, and an improvement in SLEDAI descriptors is captured when a manifestation has completely resolved. The SRI and SLEDAI share the same disadvantages by missing the signal toward improvement. The SRI original organ scoring is not weighted by severity but by overall impact on disease.

##### Administrative burden

SRI and SRI-50 require training for optimal performance. Data retrieval forms have been developed and posted on dedicated web sites that offer training and examination modules for physicians and trainees.

##### Responsiveness to patient concerns

The SRI reported clinically meaningful improvement that correlated with all domain scores of SF-36 and the FACIT (Functional Assessment of Chronic Illness Therapy) fatigue scores [32]. In addition, the partial improvement measured by SRI-50 was felt by the clinicians to reflect a clinically important improvement. Nonetheless, the effect size of the original SRI (4-point drop in SLEDAI) is at best modest. It remains unclear whether this effect size is the optimal discriminatory endpoint that reflects responsiveness to patient concerns.

### The BILAG-Based Composite Lupus Assessment

The BILAG-Based Composite Lupus Assessment (BICLA) is a composite index that was originally derived by expert consensus of disease activity indices [33]. The BICLA response was the primary endpoint in the EMBLEM (Study of Epratuzumab in Serologically-positive Systemic Lupus Erythematosus (SLE) Patients With Active Disease) (NCT00624351), a 12 -week multicenter, phase IIb randomized double-blind placebo-controlled trial that assessed the efficacy and safety of epratuzumab in patients with moderate-to-severe SLE disease activity. Requirement for the BICLA response were: (1) BILAG-2004 improvement (all A scores at baseline improved to B/C/D, and all B scores improved to C or D); (2) no worsening in disease activity (no new BILAG A or more than one new BILAG B score); (3) no worsening of total SLEDAI-2 K score from baseline; (4) no significant deterioration (<10 % worsening) in physician’s global assessment; and (5) no treatment failure (initiation of non-protocol treatment) [34].

#### Psychometric information

The SLEDAI and BILAG are the key drivers of the SRI and the BICLA responders, and their sensitivity and responsiveness to change have been shown in comparative studies with other lupus activity measurements. Direct comparison of the SRI and the BICLA psychometric properties requires caution because of the different methodologies employed in the development and evaluation of the clinical parameters contained in both indices.

Direct comparison between the BICLA and the SRI composite endpoints has been addressed in few studies [35, 36]. Disagreement between BICLA and SRI is observed, and is driven by a scoring issue. BICLA criteria require a stringent response in all body systems that are involved at baseline, and no new flares in the remaining body system are allowed. This is compared with SRI, in which a subject could qualify as a responder when a feature of SLEDAI resolves, while other features (if present at baseline) stayed the same or worsened slightly.

A similar analysis was applied to the data in the Biomarkers of Lupus Disease (BOLD) study [36]. The BICLA and SRI were compared with a simpler BOLD study response criteria minimally defined by either a drop of at least one BILAG grade or at least a 4-point reduction in SLEDAI from baseline. BICLA was found to be superior to SRI in detecting improvement and less likely to pick up flare visit.

#### Administrative burden

BICLA is completed by a physician. A computer program is needed to calculate categorical or numerical scoring. Formal training of raters and a well-defined glossary are required to ensure the optimal performance.

#### Responsiveness to patient concerns

Epratuzumab treatment in the EMBLEM trial using the BICLA showed clinically meaningful and sustained improvements in patient and physician global assessments of disease activity, SF-36 and quality of life, and reductions in corticosteroid doses.

## Organ-specific outcome measures

Several lupus nephritis outcome measures have been proposed and implemented, including the quantitative change in urinary sediments, proteinuria, renal function, and adverse events with histopathology serving as a case definition and, when relevant, as an additional endpoint [37, 38]. Composite outcomes defined as improvement (complete response, partial response, or no response), reduction in renal flares, or increase in time to flare were adopted [39, 40]. A *post hoc* analysis of the data from the abatacept trial highlighted the response criteria from different nephritis trials [41]. Given the current poor prognosis for the renal disease, a consensus-derived lupus nephritis response document is urgently needed, generally on the basis of trivial administrative burden.

Similarly, the Cutaneous Lupus Erythematosus Disease Area and Severity Index (CLASI) and the revised CLASI, a comprehensive tool for assessment of disease activity and damage in cutaneous lupus, were shown to be valid, reliable, and sensitive to changes in disease activity [42–44]. Nonetheless, CLASI scoring is heavily influenced by the number of areas involved rather than the coverage of skin within each area, and higher weighting of visible areas tends to cause greater patient impairment. CLASI has demonstrated validity by dermatologists and rheumatologists and is responsive to patient-reported measures.

## Measuring SLE flares

Several studies have attempted to define flare, including time to flare, numbers of flares, and severity of flares. Optimal SLEDAI cutoffs for active disease and flare, based on a physician’s expert opinion, have been examined. Flare was defined as a 4-point increase in SLEDAI-2 K.

The SELENA-SLEDAI Flare Index (SFI), developed by the SELENA trials, is a composite outcome of SELENA-SLEDAI; mild, moderate, and severe flares; and the PGA of disease activity [7]. The revised SFI suggests specific clinical manifestations for each organ system and categorizes flares into mild, moderate, and severe on the basis of the treatment decision.

### The BILAG-2004 flare index

A composite BILAG-2004 flare index has been proposed with flares determined by the number of systems scoring A or B due to items recorded as new or worse [45]. A severe flare is defined as the occurrence of at least one such A score, a moderate flare as the occurrence of at least two such B scores, and a mild flare as the occurrence of one B or at least three such C scores in separate systems.

Guidelines for scoring of worsening in the renal and hematologic domains are not yet available. Contrary to the SFI, selection of treatment does not override the clinical descriptors chosen.

When compared with the SFI and the physician’s global assessment, the BILAG-2004 flare index showed the highest inter-rater reliability. There was good agreement between the indices when distinguishing flares and no flares but much less consistency with mild to moderate flares. A definition of flare has been proposed by a formal Delphi consensus process. The clinical judgment of the physician is emphasized for determination of a flare, which indicates a significant change in disease activity [46].

## Conclusions

The judgment of whether a patient with SLE is better or worse is a central question in patient management. After the failure of several clinical trials of biologic therapy in SLE, the management of SLE today remains an art rather than a science. The objective is to improve disease state or at least fend off its deterioration, be accurate in defining disease activity and flare state, and employ evidence-based and clinically meaningful response criteria measured with valid and reproducible instruments that are sensitive to change and responsive to a patient’s concerns.

Pitfalls in lupus disease activity measures have had a significant impact on the interpretation of study outcomes. Many of the clinical trials were overpowered or underpowered and had complex and multiple outcome measures. Devised in the 1990s, these metrics were never intended for use in clinical trials. This underlines the importance for improving these instruments and optimizing on the composite indices for ascertainment of disease activity. Nonetheless, in the absence of a biomarker-based gold standard against which to gauge improvement or flare, selection of SLE disease endpoints should be defined and tailored to the outcome of interest. Moreover, the exact choice of disease activity-measuring instruments should be governed by the purpose for which they are required in clinical research. They should be simple, reliable, and valid with reduced administrative burden, which may integrate elements for enhanced responsiveness to patient concerns by using the platform by the published FDA guidance document. It is essential to make certain that disease activity measures are being applied consistently and uniformly through proper and simple training, given the potential complexity of patients with different SLE manifestations.

## Note

This article is part of the series ‘*Measuring meaningful change in lupus clinical trials*’, edited by Matthew Liang and Chan-Bum Choi. Other articles in this series can be found at http://arthritis-research.com/series/trials

## Abbreviations

BICLA: British Isles Lupus Assessment Group-Based Composite Lupus Assessment
BILAG: British Isles Lupus Assessment Group
BLIPS: British Lupus Integrated Prospective System
BOLD: Biomarkers of Lupus Disease
CI: confidence interval
CLASI: Cutaneous Lupus Erythematosus Disease Area and Severity Index
EMBLEM: Study of Epratuzumab in Serologically-positive Systemic Lupus Erythematosus (SLE) Patients With Active Disease
FDA: US Food and Drug Administration
HRQOL: Health-related quality of life
ICC: Intraclass correlation coefficient
PGA: Physician-rated disease activity
SELENA: Safety of Estrogens in Lupus National Assessment
SF-36: 36-Item Short Form Health Survey
SFI: The SELENA-SLEDAI Flare Index
SLAM: Systemic Lupus Activity Measure
SLAM-R: Systemic Lupus Activity Measure-Revised
SLE: Systemic lupus erythematosus
SLEDAI: Systemic Lupus Erythematosus Disease Activity Index
SLEDAI-2 K: Systemic Lupus Erythematosus Disease Activity Index-2000
SRI: Systemic Lupus Erythematosus Responder Index
SRI-50: SLEDAI-2000 Responder Index 50
